# [μ-Bis(diphenyl­arsino)methane-1:2κ^2^ *As*:*As*′]nona­carbonyl-1κ^3^ *C*,2κ^3^ *C*,3κ^3^ *C*-[tris­(4-methyl­phen­yl)­phosphine-3κ*P*]-*triangulo*-triruthenium(0)

**DOI:** 10.1107/S1600536809049940

**Published:** 2009-12-09

**Authors:** Omar bin Shawkataly, Imthyaz Ahmed Khan, Chin Sing Yeap, Hoong-Kun Fun

**Affiliations:** a Chemical Sciences Programme, School of Distance Education, Universiti Sains Malaysia, 11800 USM, Penang, Malaysia; b X-ray Crystallography Unit, School of Physics, Universiti Sains Malaysia, 11800 USM, Penang, Malaysia

## Abstract

In the title *triangulo*-triruthenium compound, [Ru_3_(C_25_H_22_As_2_)(C_21_H_21_P)(CO)_9_], the bis­(diphenyl­arsino)methane ligand bridges a Ru—Ru bond and the monodentate phosphine ligand bonds to the third Ru atom. Both the phosphine and arsine ligands are equatorial with respect to the Ru_3_ triangle. Additionally, each Ru atom carries one equatorial and two axial terminal carbonyl ligands. The three phenyl rings of the phosphine make dihedral angles of 86.89 (19), 82.1 (2) and 63.0 (2)° with each other. The dihedral angles between the two phenyl rings are 73.8 (2) and 82.2 (3)° for the two diphenyl­arsino groups. An intra­molecular C—H⋯O hydrogen bond stabilizes the mol­ecular structure. In the crystal packing, mol­ecules are linked into chains down the *b* axis *via* inter­molecular C—H⋯O hydrogen bonds.

## Related literature

For general background to *triangulo*-triruthenium derivatives, see: Bruce *et al.* (1985 ▶, 1988*a* ▶,*b* ▶); Shawkataly *et al.* (1998 ▶, 2004 ▶, 2009 ▶). For related structures, see: Shawkataly *et al.* (2009 ▶). For the synthesis of μ-bis­(diphenylarsino)methanedecacarbonyl­triruthenium(0), see: Bruce *et al.* (1983 ▶). For the stability of the temperature controller used in the data collection, see: Cosier & Glazer (1986 ▶).
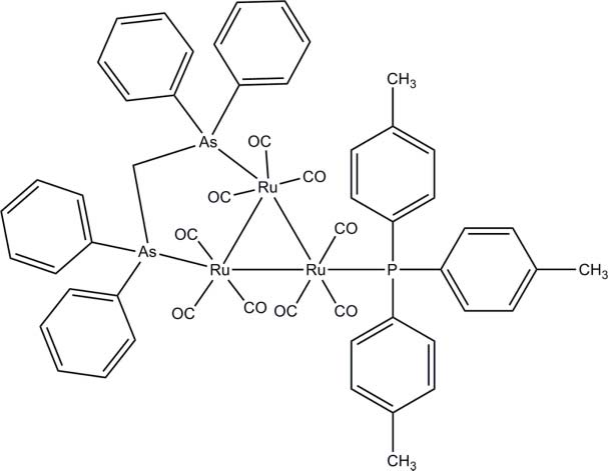


## Experimental

### 

#### Crystal data

[Ru_3_(C_25_H_22_As_2_)(C_21_H_21_P)(CO)_9_]
*M* *_r_* = 1331.91
Monoclinic, 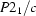

*a* = 16.2585 (2) Å
*b* = 16.9247 (2) Å
*c* = 19.6900 (2) Å
β = 98.680 (1)°
*V* = 5356.05 (11) Å^3^
*Z* = 4
Mo *K*α radiation
μ = 2.15 mm^−1^
*T* = 296 K
0.21 × 0.21 × 0.03 mm

#### Data collection

Bruker SMART APEXII CCD area-detector diffractometer
Absorption correction: multi-scan (**SADABS**; Bruker, 2005 ▶) *T*_min_ = 0.665, *T*_max_ = 0.933
63277 measured reflections
15703 independent reflections
9536 reflections with *I* > 2σ(*I*)
*R*_int_ = 0.063

#### Refinement

*R*[*F* ^2^ > 2σ(*F* ^2^)] = 0.049
*wR*(*F* ^2^) = 0.096
*S* = 1.00
15703 reflections
634 parameters
H-atom parameters constrained
Δρ_max_ = 0.60 e Å^−3^
Δρ_min_ = −0.41 e Å^−3^

### 

Data collection: *APEX2* (Bruker, 2005 ▶); cell refinement: *SAINT* (Bruker, 2005 ▶); data reduction: *SAINT*; program(s) used to solve structure: *SHELXTL* (Sheldrick, 2008 ▶); program(s) used to refine structure: *SHELXTL*; molecular graphics: *SHELXTL*; software used to prepare material for publication: *SHELXTL* and *PLATON* (Spek, 2009 ▶).

## Supplementary Material

Crystal structure: contains datablocks global, I. DOI: 10.1107/S1600536809049940/sj2681sup1.cif

Structure factors: contains datablocks I. DOI: 10.1107/S1600536809049940/sj2681Isup2.hkl

Additional supplementary materials:  crystallographic information; 3D view; checkCIF report

## Figures and Tables

**Table 1:** Hydrogen-bond geometry (Å, °)

*D*—H⋯*A*	*D*—H	H⋯*A*	*D*⋯*A*	*D*—H⋯*A*
C27—H27*A*⋯O9	0.93	2.57	3.317 (5)	138
C54—H54*A*⋯O2^i^	0.96	2.60	3.306 (6)	131

Symmetry code: (i) 
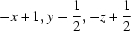
.

**Fig. 1.**
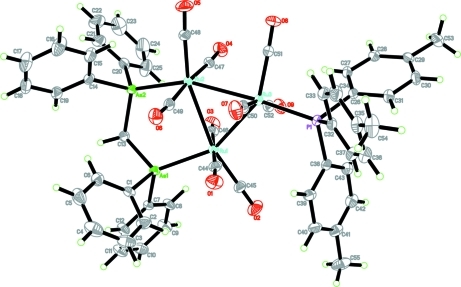
The molecular structure of the title compound with 20% probability ellipsoids for non-H atoms.

**Fig. 2.**
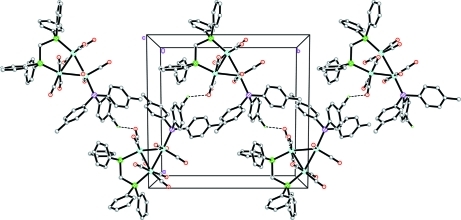
The crystal packing of the title compound, viewed down the c axis, showing the molecules linked along b. Hydrogen atoms that are not involved in the hydrogen-bonding (dashed lines) have been omitted for clarity.

## supplementary crystallographic information

### Comment

*Triangulo*-triruthenium clusters are known for their interesting structural variations and related catalytic activity. A large number of substituted derivatives, Ru_3_(CO)_12-n_*L*_n_ (*L*= group 15 ligand) have been reported (Bruce *et al.*, 1985, 1988a,b). As part of our study on the substitution of transition metal-carbonyl clusters with mixed-ligand complexes, we have published several structures of *triangulo*-triruthenium-carbonyl clusters containing mixed P/As and P/Sb ligands (Shawkataly *et al.*, 1998, 2004, 2009). Herein we report the synthesis and structure of Ru_3_(C_21_H_21_P)(C_25_H_22_As_2_)(CO)_9_.

The bond lengths and angles of title compound (Fig. 1) are comparable to those in related structures (Shawkataly *et al.*, 2009). The bis(diphenylarsino)methane ligand bridges the Ru1—Ru2 bond and the monodentate phosphine ligand bonds to the Ru3 atom. Both the phosphine and arsine ligands are equatorial with respect to the Ru_3_ triangle. Additionally, each Ru atom carries one equatorial and two axial terminal carbonyl ligands. The three phosphine substituted phenyl rings make dihedral angles (C26–C31/C32–C37, C26–C31/C38–C43 and C32–C37/C38–C43) of 86.89 (19), 82.1 (2) and 63.0 (2)° with each other respectively. The dihedral angles between the two phenyl rings (C1–C6/C7–C12 and C14–C19/C20–C25) are 73.8 (2) and 82.2 (3)° for the two diphenylarsino groups respectively.

An intramolecular C27—H27A···O9 hydrogen bond stabilizes the molecular structure. In the crystal packing (Fig. 2), the molecules are linked together into chains *via* intermolecular C54—H54A···O2 hydrogen bonds along the *b* axis.

### Experimental

All manipulations were performed under a dry oxygen-free dinitrogen atmosphere using standard Schlenk techniques, all solvents were dried over sodium and distilled from sodium benzophenone ketyl under nitrogen. Tris(4-methylphenyl)phosphine (Maybridge) was used as received and µ-bis(diphenylarsino)methanedecacarbonyltriruthenium(0) (Bruce *et al.*, 1983) was prepared by a previously reported procedure. The title compound was obtained by refluxing equimolar quantities of Ru_3_(CO)_10_(µ-Ph_2_AsCH_2_AsPh_2_) (105.5 mg, 0.1 mmol) and tris(4-methylphenyl)phosphine (30.4 mg, 0.1 mmol) in hexane under a nitrogen atmosphere. Crystals suitable for X-ray diffraction were grown by slow solvent / solvent diffusion of CH_3_OH into CH_2_Cl_2_.

### Refinement

All hydrogen atoms were positioned geometrically and refined using a riding model with C—H = 0.93–0.97 Å and *U*_iso_(H) = 1.2 or 1.5 *U*_eq_(C). A rotating group model was used for the methyl groups.

### Crystal data

**Table:**

[Ru_3_(C_25_H_22_As_2_)(C_21_H_21_P)(CO)_9_]	*F*(000) = 2632
*M**_r_* = 1331.91	*D*_x_ = 1.652 Mg m^−^^3^
Monoclinic, *P*2_1_/*c*	Mo *K*α radiation, λ = 0.71073 Å
Hall symbol: -P 2ybc	Cell parameters from 9940 reflections
*a* = 16.2585 (2) Å	θ = 2.3–26.0°
*b* = 16.9247 (2) Å	µ = 2.15 mm^−^^1^
*c* = 19.6900 (2) Å	*T* = 296 K
β = 98.680 (1)°	Plate, red
*V* = 5356.05 (11) Å^3^	0.21 × 0.21 × 0.03 mm
*Z* = 4	

### Data collection

**Table:**

Bruker SMART APEXII CCD area-detector diffractometer	15703 independent reflections
Radiation source: fine-focus sealed tube	9536 reflections with *I* > 2σ(*I*)
graphite	*R*_int_ = 0.063
φ and ω scans	θ_max_ = 30.1°, θ_min_ = 1.6°
Absorption correction: multi-scan (*SADABS*; Bruker, 2005)	*h* = −22→22
*T*_min_ = 0.665, *T*_max_ = 0.933	*k* = −18→23
63277 measured reflections	*l* = −27→27

### Refinement

**Table:**

Refinement on *F*^2^	Primary atom site location: structure-invariant direct methods
Least-squares matrix: full	Secondary atom site location: difference Fourier map
*R*[*F*^2^ > 2σ(*F*^2^)] = 0.049	Hydrogen site location: inferred from neighbouring sites
*wR*(*F*^2^) = 0.096	H-atom parameters constrained
*S* = 1.00	*w* = 1/[σ^2^(*F*_o_^2^) + (0.0397*P*)^2^] where *P* = (*F*_o_^2^ + 2*F*_c_^2^)/3
15703 reflections	(Δ/σ)_max_ < 0.001
634 parameters	Δρ_max_ = 0.60 e Å^−^^3^
0 restraints	Δρ_min_ = −0.40 e Å^−^^3^

### Special details

**Table:**

Geometry. All e.s.d.'s (except the e.s.d. in the dihedral angle between two l.s. planes) are estimated using the full covariance matrix. The cell e.s.d.'s are taken into account individually in the estimation of e.s.d.'s in distances, angles and torsion angles; correlations between e.s.d.'s in cell parameters are only used when they are defined by crystal symmetry. An approximate (isotropic) treatment of cell e.s.d.'s is used for estimating e.s.d.'s involving l.s. planes.
Refinement. Refinement of *F*^2^ against ALL reflections. The weighted *R*-factor *wR* and goodness of fit *S* are based on *F*^2^, conventional *R*-factors *R* are based on *F*, with *F* set to zero for negative *F*^2^. The threshold expression of *F*^2^ > σ(*F*^2^) is used only for calculating *R*-factors(gt) *etc*. and is not relevant to the choice of reflections for refinement. *R*-factors based on *F*^2^ are statistically about twice as large as those based on *F*, and *R*- factors based on ALL data will be even larger.

### Fractional atomic coordinates and isotropic or equivalent isotropic displacement parameters (Å^2^)

**Table:**

	*x*	*y*	*z*	*U*_iso_*/*U*_eq_	
Ru1	0.234430 (18)	0.082258 (18)	0.290394 (17)	0.03903 (8)	
Ru2	0.095682 (17)	0.005658 (17)	0.211088 (16)	0.03574 (8)	
Ru3	0.250620 (18)	−0.075182 (17)	0.238649 (17)	0.03759 (8)	
As1	0.16874 (2)	0.21116 (2)	0.26830 (2)	0.03705 (10)	
As2	−0.00352 (2)	0.11309 (2)	0.21734 (2)	0.03700 (10)	
P1	0.38304 (6)	−0.13329 (6)	0.26418 (5)	0.0402 (2)	
O1	0.3348 (2)	0.10979 (19)	0.17289 (18)	0.0784 (10)	
O2	0.3831 (2)	0.1220 (2)	0.3968 (2)	0.0934 (12)	
O3	0.1375 (2)	0.05987 (18)	0.41083 (17)	0.0741 (10)	
O4	0.0457 (2)	−0.08636 (18)	0.33265 (19)	0.0782 (11)	
O5	−0.0027 (2)	−0.1159 (2)	0.1199 (2)	0.0924 (12)	
O6	0.1513 (2)	0.09138 (18)	0.08889 (17)	0.0699 (9)	
O7	0.2824 (2)	−0.0409 (2)	0.09116 (17)	0.0756 (10)	
O8	0.1550 (2)	−0.22473 (19)	0.19949 (19)	0.0814 (11)	
O9	0.2461 (2)	−0.08632 (17)	0.39289 (17)	0.0697 (9)	
C1	0.1912 (2)	0.2681 (2)	0.1877 (2)	0.0445 (10)	
C2	0.2732 (3)	0.2890 (3)	0.1858 (3)	0.0652 (13)	
H2A	0.3141	0.2753	0.2221	0.078*	
C3	0.2948 (4)	0.3300 (3)	0.1304 (3)	0.0791 (16)	
H3A	0.3500	0.3436	0.1296	0.095*	
C4	0.2357 (4)	0.3504 (3)	0.0775 (3)	0.0845 (18)	
H4A	0.2504	0.3785	0.0406	0.101*	
C5	0.1538 (4)	0.3297 (3)	0.0780 (3)	0.0817 (16)	
H5A	0.1132	0.3430	0.0412	0.098*	
C6	0.1326 (3)	0.2889 (3)	0.1338 (2)	0.0616 (12)	
H6A	0.0773	0.2755	0.1345	0.074*	
C7	0.1927 (2)	0.2926 (2)	0.3382 (2)	0.0405 (9)	
C8	0.2062 (3)	0.2709 (2)	0.4064 (2)	0.0538 (11)	
H8A	0.2058	0.2176	0.4177	0.065*	
C9	0.2204 (3)	0.3265 (3)	0.4583 (2)	0.0671 (14)	
H9A	0.2289	0.3107	0.5041	0.081*	
C10	0.2217 (3)	0.4046 (3)	0.4418 (3)	0.0668 (14)	
H10A	0.2314	0.4425	0.4763	0.080*	
C11	0.2086 (3)	0.4272 (3)	0.3744 (3)	0.0675 (14)	
H11A	0.2099	0.4806	0.3634	0.081*	
C12	0.1936 (3)	0.3718 (2)	0.3225 (2)	0.0578 (12)	
H12A	0.1841	0.3881	0.2769	0.069*	
C13	0.0474 (2)	0.2089 (2)	0.2622 (2)	0.0416 (9)	
H13A	0.0243	0.2548	0.2367	0.050*	
H13B	0.0330	0.2123	0.3082	0.050*	
C14	−0.0632 (2)	0.1517 (2)	0.1305 (2)	0.0448 (10)	
C15	−0.0724 (3)	0.1023 (3)	0.0737 (3)	0.0661 (13)	
H15A	−0.0508	0.0513	0.0779	0.079*	
C16	−0.1131 (4)	0.1278 (4)	0.0115 (3)	0.0925 (19)	
H16A	−0.1193	0.0936	−0.0259	0.111*	
C17	−0.1448 (4)	0.2033 (4)	0.0036 (3)	0.0920 (19)	
H17A	−0.1715	0.2206	−0.0390	0.110*	
C18	−0.1363 (3)	0.2525 (3)	0.0599 (3)	0.0763 (15)	
H18A	−0.1572	0.3037	0.0554	0.092*	
C19	−0.0973 (3)	0.2266 (3)	0.1227 (2)	0.0560 (11)	
H19A	−0.0937	0.2599	0.1605	0.067*	
C20	−0.0938 (2)	0.0935 (2)	0.2700 (2)	0.0501 (11)	
C21	−0.1754 (3)	0.0953 (3)	0.2391 (3)	0.0676 (14)	
H21A	−0.1886	0.1111	0.1935	0.081*	
C22	−0.2379 (3)	0.0736 (4)	0.2756 (4)	0.095 (2)	
H22A	−0.2931	0.0742	0.2545	0.114*	
C23	−0.2189 (4)	0.0514 (4)	0.3419 (4)	0.113 (3)	
H23A	−0.2612	0.0352	0.3657	0.136*	
C24	−0.1385 (4)	0.0523 (4)	0.3747 (4)	0.123 (3)	
H24A	−0.1261	0.0390	0.4209	0.148*	
C25	−0.0748 (3)	0.0737 (3)	0.3370 (3)	0.0853 (18)	
H25A	−0.0197	0.0743	0.3583	0.102*	
C26	0.3884 (2)	−0.2298 (2)	0.3075 (2)	0.0416 (9)	
C27	0.3279 (2)	−0.2538 (2)	0.3465 (2)	0.0529 (11)	
H27A	0.2810	−0.2226	0.3474	0.064*	
C28	0.3370 (3)	−0.3233 (2)	0.3841 (2)	0.0581 (12)	
H28A	0.2967	−0.3370	0.4108	0.070*	
C29	0.4040 (3)	−0.3724 (2)	0.3827 (2)	0.0545 (11)	
C30	0.4632 (3)	−0.3498 (2)	0.3433 (2)	0.0584 (12)	
H30A	0.5088	−0.3825	0.3412	0.070*	
C31	0.4564 (3)	−0.2794 (2)	0.3066 (2)	0.0520 (11)	
H31A	0.4979	−0.2652	0.2812	0.062*	
C32	0.4366 (2)	−0.1552 (2)	0.1903 (2)	0.0434 (9)	
C33	0.3920 (3)	−0.1915 (2)	0.1337 (2)	0.0546 (11)	
H33A	0.3360	−0.2028	0.1333	0.066*	
C34	0.4297 (3)	−0.2114 (3)	0.0773 (2)	0.0644 (13)	
H34A	0.3983	−0.2353	0.0394	0.077*	
C35	0.5131 (3)	−0.1962 (3)	0.0764 (2)	0.0579 (12)	
C36	0.5573 (3)	−0.1617 (3)	0.1335 (2)	0.0596 (12)	
H36A	0.6138	−0.1519	0.1344	0.072*	
C37	0.5202 (2)	−0.1409 (2)	0.1898 (2)	0.0533 (11)	
H37A	0.5519	−0.1171	0.2276	0.064*	
C38	0.4591 (2)	−0.0747 (2)	0.3211 (2)	0.0416 (9)	
C39	0.4872 (3)	−0.0032 (2)	0.2982 (2)	0.0546 (11)	
H39A	0.4691	0.0130	0.2533	0.065*	
C40	0.5415 (3)	0.0441 (3)	0.3412 (3)	0.0585 (12)	
H40A	0.5603	0.0911	0.3244	0.070*	
C41	0.5683 (3)	0.0229 (3)	0.4083 (2)	0.0578 (12)	
C42	0.5398 (3)	−0.0477 (3)	0.4309 (2)	0.0592 (12)	
H42A	0.5572	−0.0635	0.4760	0.071*	
C43	0.4858 (2)	−0.0957 (2)	0.3878 (2)	0.0491 (11)	
H43A	0.4675	−0.1429	0.4045	0.059*	
C44	0.2951 (3)	0.0928 (2)	0.2144 (2)	0.0522 (11)	
C45	0.3291 (3)	0.1071 (3)	0.3549 (3)	0.0582 (12)	
C46	0.1713 (3)	0.0653 (2)	0.3637 (2)	0.0518 (11)	
C47	0.0670 (2)	−0.0503 (2)	0.2894 (2)	0.0499 (11)	
C48	0.0325 (2)	−0.0683 (3)	0.1537 (2)	0.0525 (11)	
C49	0.1344 (2)	0.0597 (2)	0.1357 (2)	0.0475 (10)	
C50	0.2690 (3)	−0.0515 (2)	0.1462 (2)	0.0494 (10)	
C51	0.1925 (2)	−0.1692 (3)	0.2141 (2)	0.0505 (11)	
C52	0.2446 (3)	−0.0733 (2)	0.3344 (2)	0.0505 (11)	
C53	0.4136 (3)	−0.4481 (3)	0.4251 (3)	0.0830 (16)	
H53A	0.4712	−0.4558	0.4433	0.124*	
H53B	0.3940	−0.4921	0.3964	0.124*	
H53C	0.3818	−0.4439	0.4622	0.124*	
C54	0.5545 (3)	−0.2172 (3)	0.0149 (2)	0.0850 (18)	
H54A	0.6021	−0.2500	0.0294	0.127*	
H54B	0.5718	−0.1698	−0.0056	0.127*	
H54C	0.5157	−0.2452	−0.0182	0.127*	
C55	0.6253 (3)	0.0748 (3)	0.4567 (3)	0.095 (2)	
H55A	0.6557	0.1092	0.4307	0.142*	
H55B	0.6635	0.0424	0.4865	0.142*	
H55C	0.5928	0.1059	0.4837	0.142*	

### Atomic displacement parameters (Å^2^)

**Table:**

	*U*^11^	*U*^22^	*U*^33^	*U*^12^	*U*^13^	*U*^23^
Ru1	0.03420 (15)	0.03399 (18)	0.04736 (19)	−0.00229 (13)	0.00114 (15)	−0.00603 (15)
Ru2	0.02854 (13)	0.03101 (17)	0.04748 (18)	−0.00082 (12)	0.00511 (13)	−0.00357 (14)
Ru3	0.03213 (15)	0.03246 (17)	0.04644 (19)	0.00260 (12)	0.00032 (14)	−0.00174 (14)
As1	0.0377 (2)	0.0325 (2)	0.0409 (2)	−0.00439 (16)	0.00575 (18)	−0.00314 (17)
As2	0.03049 (18)	0.0332 (2)	0.0480 (2)	0.00017 (16)	0.00820 (18)	−0.00228 (18)
P1	0.0329 (5)	0.0406 (6)	0.0455 (6)	0.0030 (4)	0.0012 (5)	0.0006 (5)
O1	0.072 (2)	0.079 (2)	0.093 (3)	−0.0106 (18)	0.041 (2)	−0.008 (2)
O2	0.070 (2)	0.104 (3)	0.093 (3)	0.004 (2)	−0.031 (2)	−0.038 (2)
O3	0.093 (3)	0.068 (2)	0.066 (2)	−0.0084 (18)	0.028 (2)	−0.0057 (17)
O4	0.085 (2)	0.064 (2)	0.091 (3)	−0.0072 (18)	0.031 (2)	0.0289 (19)
O5	0.074 (2)	0.076 (3)	0.120 (3)	−0.017 (2)	−0.006 (2)	−0.046 (2)
O6	0.074 (2)	0.075 (2)	0.064 (2)	0.0058 (17)	0.0215 (19)	0.0193 (18)
O7	0.097 (3)	0.076 (2)	0.057 (2)	0.0244 (19)	0.020 (2)	0.0140 (18)
O8	0.079 (2)	0.048 (2)	0.109 (3)	−0.0221 (18)	−0.014 (2)	−0.005 (2)
O9	0.099 (3)	0.056 (2)	0.053 (2)	0.0103 (17)	0.008 (2)	0.0068 (16)
C1	0.050 (2)	0.033 (2)	0.051 (3)	−0.0051 (18)	0.012 (2)	−0.0029 (19)
C2	0.060 (3)	0.072 (3)	0.065 (3)	−0.016 (2)	0.014 (3)	0.006 (3)
C3	0.086 (4)	0.078 (4)	0.079 (4)	−0.027 (3)	0.034 (4)	0.002 (3)
C4	0.122 (5)	0.074 (4)	0.065 (4)	−0.022 (4)	0.039 (4)	0.007 (3)
C5	0.114 (5)	0.069 (4)	0.059 (3)	0.005 (3)	0.001 (3)	0.017 (3)
C6	0.060 (3)	0.062 (3)	0.062 (3)	−0.001 (2)	0.007 (3)	0.011 (3)
C7	0.043 (2)	0.032 (2)	0.045 (2)	−0.0035 (17)	0.0051 (19)	−0.0045 (18)
C8	0.076 (3)	0.035 (2)	0.049 (3)	−0.006 (2)	0.006 (2)	−0.005 (2)
C9	0.104 (4)	0.054 (3)	0.044 (3)	−0.008 (3)	0.014 (3)	−0.012 (2)
C10	0.088 (4)	0.055 (3)	0.060 (3)	−0.014 (3)	0.016 (3)	−0.023 (3)
C11	0.099 (4)	0.037 (3)	0.067 (3)	−0.011 (2)	0.016 (3)	−0.007 (2)
C12	0.079 (3)	0.040 (3)	0.054 (3)	−0.003 (2)	0.007 (3)	−0.004 (2)
C13	0.0393 (19)	0.039 (2)	0.047 (2)	0.0029 (16)	0.0079 (19)	−0.0054 (18)
C14	0.0347 (19)	0.050 (3)	0.049 (2)	0.0002 (18)	0.0025 (19)	−0.001 (2)
C15	0.061 (3)	0.070 (3)	0.062 (3)	0.019 (2)	−0.009 (3)	−0.016 (3)
C16	0.090 (4)	0.123 (5)	0.057 (3)	0.032 (4)	−0.014 (3)	−0.028 (4)
C17	0.092 (4)	0.116 (5)	0.062 (4)	0.035 (4)	−0.006 (3)	0.006 (4)
C18	0.074 (3)	0.071 (4)	0.076 (4)	0.020 (3)	−0.013 (3)	0.010 (3)
C19	0.055 (3)	0.048 (3)	0.062 (3)	0.008 (2)	−0.002 (2)	−0.006 (2)
C20	0.044 (2)	0.041 (2)	0.070 (3)	0.0045 (18)	0.023 (2)	0.004 (2)
C21	0.042 (2)	0.067 (3)	0.097 (4)	−0.006 (2)	0.024 (3)	−0.005 (3)
C22	0.047 (3)	0.115 (5)	0.128 (6)	−0.013 (3)	0.029 (4)	−0.005 (4)
C23	0.088 (5)	0.119 (5)	0.151 (7)	0.018 (4)	0.077 (5)	0.040 (5)
C24	0.099 (5)	0.164 (7)	0.120 (6)	0.044 (5)	0.060 (5)	0.069 (5)
C25	0.057 (3)	0.122 (5)	0.082 (4)	0.021 (3)	0.027 (3)	0.033 (3)
C26	0.040 (2)	0.034 (2)	0.048 (2)	0.0044 (16)	−0.0027 (19)	−0.0037 (18)
C27	0.039 (2)	0.044 (3)	0.074 (3)	0.0042 (19)	0.004 (2)	0.010 (2)
C28	0.047 (2)	0.046 (3)	0.079 (3)	−0.004 (2)	0.004 (2)	0.012 (2)
C29	0.058 (3)	0.036 (2)	0.064 (3)	−0.004 (2)	−0.010 (2)	0.001 (2)
C30	0.059 (3)	0.046 (3)	0.066 (3)	0.022 (2)	−0.002 (2)	−0.003 (2)
C31	0.049 (2)	0.053 (3)	0.054 (3)	0.010 (2)	0.008 (2)	0.000 (2)
C32	0.044 (2)	0.042 (2)	0.042 (2)	0.0084 (18)	0.0006 (19)	0.0015 (19)
C33	0.048 (2)	0.054 (3)	0.060 (3)	0.008 (2)	0.000 (2)	−0.005 (2)
C34	0.073 (3)	0.061 (3)	0.056 (3)	0.015 (2)	−0.002 (3)	−0.011 (2)
C35	0.066 (3)	0.059 (3)	0.049 (3)	0.023 (2)	0.007 (2)	0.007 (2)
C36	0.050 (2)	0.073 (3)	0.056 (3)	0.012 (2)	0.009 (2)	0.004 (3)
C37	0.045 (2)	0.063 (3)	0.050 (3)	0.006 (2)	0.002 (2)	−0.001 (2)
C38	0.0327 (18)	0.044 (2)	0.049 (2)	0.0033 (16)	0.0085 (18)	−0.0028 (19)
C39	0.046 (2)	0.056 (3)	0.059 (3)	−0.008 (2)	0.002 (2)	0.000 (2)
C40	0.044 (2)	0.057 (3)	0.076 (3)	−0.014 (2)	0.013 (2)	−0.010 (3)
C41	0.041 (2)	0.074 (3)	0.061 (3)	0.000 (2)	0.012 (2)	−0.024 (3)
C42	0.058 (3)	0.073 (3)	0.045 (3)	0.007 (2)	0.002 (2)	−0.012 (2)
C43	0.050 (2)	0.053 (3)	0.046 (2)	0.005 (2)	0.012 (2)	−0.002 (2)
C44	0.043 (2)	0.049 (3)	0.063 (3)	−0.0051 (19)	0.003 (2)	−0.013 (2)
C45	0.047 (2)	0.057 (3)	0.067 (3)	0.002 (2)	−0.003 (2)	−0.016 (2)
C46	0.058 (3)	0.040 (3)	0.056 (3)	−0.0021 (19)	0.005 (2)	−0.004 (2)
C47	0.042 (2)	0.038 (2)	0.070 (3)	0.0010 (18)	0.008 (2)	0.000 (2)
C48	0.040 (2)	0.047 (3)	0.069 (3)	0.0006 (19)	0.005 (2)	−0.010 (2)
C49	0.040 (2)	0.046 (3)	0.057 (3)	0.0082 (18)	0.006 (2)	−0.005 (2)
C50	0.047 (2)	0.042 (2)	0.056 (3)	0.0095 (19)	−0.001 (2)	−0.002 (2)
C51	0.042 (2)	0.043 (3)	0.064 (3)	0.0014 (19)	−0.003 (2)	0.004 (2)
C52	0.050 (2)	0.049 (3)	0.052 (3)	0.0040 (19)	0.005 (2)	−0.005 (2)
C53	0.092 (4)	0.047 (3)	0.104 (4)	−0.001 (3)	−0.003 (4)	0.017 (3)
C54	0.103 (4)	0.103 (4)	0.052 (3)	0.046 (3)	0.020 (3)	0.009 (3)
C55	0.073 (4)	0.121 (5)	0.092 (4)	−0.037 (3)	0.017 (3)	−0.057 (4)

### Geometric parameters (Å, °)

**Table:**

Ru1—C45	1.890 (5)	C16—C17	1.378 (7)
Ru1—C46	1.915 (4)	C16—H16A	0.9300
Ru1—C44	1.922 (4)	C17—C18	1.378 (7)
Ru1—As1	2.4391 (5)	C17—H17A	0.9300
Ru1—Ru2	2.8539 (4)	C18—C19	1.373 (6)
Ru1—Ru3	2.8790 (4)	C18—H18A	0.9300
Ru2—C48	1.884 (5)	C19—H19A	0.9300
Ru2—C47	1.925 (4)	C20—C25	1.352 (7)
Ru2—C49	1.929 (4)	C20—C21	1.374 (6)
Ru2—As2	2.4460 (4)	C21—C22	1.380 (6)
Ru2—Ru3	2.8460 (4)	C21—H21A	0.9300
Ru3—C51	1.876 (5)	C22—C23	1.348 (9)
Ru3—C52	1.904 (4)	C22—H22A	0.9300
Ru3—C50	1.931 (5)	C23—C24	1.368 (10)
Ru3—P1	2.3513 (10)	C23—H23A	0.9300
As1—C1	1.938 (4)	C24—C25	1.410 (7)
As1—C7	1.946 (4)	C24—H24A	0.9300
As1—C13	1.958 (3)	C25—H25A	0.9300
As2—C14	1.947 (4)	C26—C31	1.390 (5)
As2—C20	1.948 (3)	C26—C27	1.397 (5)
As2—C13	1.968 (4)	C27—C28	1.386 (5)
P1—C38	1.831 (4)	C27—H27A	0.9300
P1—C26	1.839 (4)	C28—C29	1.374 (6)
P1—C32	1.842 (4)	C28—H28A	0.9300
O1—C44	1.151 (5)	C29—C30	1.379 (6)
O2—C45	1.138 (5)	C29—C53	1.523 (6)
O3—C46	1.151 (4)	C30—C31	1.389 (5)
O4—C47	1.144 (5)	C30—H30A	0.9300
O5—C48	1.141 (5)	C31—H31A	0.9300
O6—C49	1.135 (5)	C32—C33	1.379 (6)
O7—C50	1.151 (5)	C32—C37	1.382 (5)
O8—C51	1.134 (5)	C33—C34	1.389 (6)
O9—C52	1.168 (5)	C33—H33A	0.9300
C1—C6	1.362 (6)	C34—C35	1.383 (6)
C1—C2	1.385 (5)	C34—H34A	0.9300
C2—C3	1.381 (6)	C35—C36	1.370 (6)
C2—H2A	0.9300	C35—C54	1.514 (6)
C3—C4	1.350 (7)	C36—C37	1.385 (5)
C3—H3A	0.9300	C36—H36A	0.9300
C4—C5	1.379 (8)	C37—H37A	0.9300
C4—H4A	0.9300	C38—C43	1.366 (5)
C5—C6	1.384 (6)	C38—C39	1.392 (5)
C5—H5A	0.9300	C39—C40	1.383 (6)
C6—H6A	0.9300	C39—H39A	0.9300
C7—C12	1.376 (5)	C40—C41	1.374 (6)
C7—C8	1.376 (5)	C40—H40A	0.9300
C8—C9	1.383 (6)	C41—C42	1.379 (6)
C8—H8A	0.9300	C41—C55	1.508 (6)
C9—C10	1.363 (6)	C42—C43	1.388 (6)
C9—H9A	0.9300	C42—H42A	0.9300
C10—C11	1.366 (6)	C43—H43A	0.9300
C10—H10A	0.9300	C53—H53A	0.9600
C11—C12	1.381 (6)	C53—H53B	0.9600
C11—H11A	0.9300	C53—H53C	0.9600
C12—H12A	0.9300	C54—H54A	0.9600
C13—H13A	0.9700	C54—H54B	0.9600
C13—H13B	0.9700	C54—H54C	0.9600
C14—C19	1.382 (5)	C55—H55A	0.9600
C14—C15	1.386 (6)	C55—H55B	0.9600
C15—C16	1.372 (7)	C55—H55C	0.9600
C15—H15A	0.9300		
			
C45—Ru1—C46	90.09 (19)	C15—C16—C17	121.0 (5)
C45—Ru1—C44	92.37 (18)	C15—C16—H16A	119.5
C46—Ru1—C44	176.30 (17)	C17—C16—H16A	119.5
C45—Ru1—As1	102.18 (14)	C16—C17—C18	118.7 (5)
C46—Ru1—As1	89.88 (12)	C16—C17—H17A	120.7
C44—Ru1—As1	92.30 (12)	C18—C17—H17A	120.7
C45—Ru1—Ru2	164.91 (15)	C19—C18—C17	120.5 (5)
C46—Ru1—Ru2	82.81 (13)	C19—C18—H18A	119.8
C44—Ru1—Ru2	94.15 (13)	C17—C18—H18A	119.8
As1—Ru1—Ru2	91.150 (15)	C18—C19—C14	121.2 (4)
C45—Ru1—Ru3	109.60 (13)	C18—C19—H19A	119.4
C46—Ru1—Ru3	102.50 (12)	C14—C19—H19A	119.4
C44—Ru1—Ru3	74.06 (12)	C25—C20—C21	120.1 (4)
As1—Ru1—Ru3	145.659 (18)	C25—C20—As2	118.8 (3)
Ru2—Ru1—Ru3	59.526 (10)	C21—C20—As2	121.0 (4)
C48—Ru2—C47	88.73 (18)	C20—C21—C22	120.1 (5)
C48—Ru2—C49	93.74 (18)	C20—C21—H21A	120.0
C47—Ru2—C49	174.99 (17)	C22—C21—H21A	120.0
C48—Ru2—As2	102.71 (12)	C23—C22—C21	120.0 (6)
C47—Ru2—As2	94.85 (11)	C23—C22—H22A	120.0
C49—Ru2—As2	88.86 (11)	C21—C22—H22A	120.0
C48—Ru2—Ru3	100.48 (12)	C22—C23—C24	121.1 (5)
C47—Ru2—Ru3	85.69 (12)	C22—C23—H23A	119.5
C49—Ru2—Ru3	89.58 (11)	C24—C23—H23A	119.5
As2—Ru2—Ru3	156.811 (17)	C23—C24—C25	118.7 (6)
C48—Ru2—Ru1	160.83 (12)	C23—C24—H24A	120.6
C47—Ru2—Ru1	92.99 (13)	C25—C24—H24A	120.6
C49—Ru2—Ru1	83.26 (13)	C20—C25—C24	120.0 (5)
As2—Ru2—Ru1	96.175 (14)	C20—C25—H25A	120.0
Ru3—Ru2—Ru1	60.675 (11)	C24—C25—H25A	120.0
C51—Ru3—C52	99.79 (18)	C31—C26—C27	117.3 (4)
C51—Ru3—C50	94.40 (18)	C31—C26—P1	120.9 (3)
C52—Ru3—C50	165.73 (17)	C27—C26—P1	121.6 (3)
C51—Ru3—P1	96.29 (12)	C28—C27—C26	120.9 (4)
C52—Ru3—P1	88.70 (13)	C28—C27—H27A	119.6
C50—Ru3—P1	91.23 (12)	C26—C27—H27A	119.6
C51—Ru3—Ru2	87.81 (12)	C29—C28—C27	121.6 (4)
C52—Ru3—Ru2	90.27 (12)	C29—C28—H28A	119.2
C50—Ru3—Ru2	88.79 (12)	C27—C28—H28A	119.2
P1—Ru3—Ru2	175.88 (3)	C28—C29—C30	117.8 (4)
C51—Ru3—Ru1	143.77 (12)	C28—C29—C53	120.9 (4)
C52—Ru3—Ru1	67.23 (12)	C30—C29—C53	121.2 (4)
C50—Ru3—Ru1	100.20 (12)	C29—C30—C31	121.5 (4)
P1—Ru3—Ru1	116.18 (3)	C29—C30—H30A	119.2
Ru2—Ru3—Ru1	59.798 (10)	C31—C30—H30A	119.2
C1—As1—C7	100.67 (16)	C30—C31—C26	120.8 (4)
C1—As1—C13	105.80 (17)	C30—C31—H31A	119.6
C7—As1—C13	98.57 (15)	C26—C31—H31A	119.6
C1—As1—Ru1	117.32 (11)	C33—C32—C37	118.0 (4)
C7—As1—Ru1	118.18 (12)	C33—C32—P1	118.3 (3)
C13—As1—Ru1	113.78 (11)	C37—C32—P1	123.6 (3)
C14—As2—C20	102.02 (17)	C32—C33—C34	120.9 (4)
C14—As2—C13	103.76 (17)	C32—C33—H33A	119.6
C20—As2—C13	101.59 (16)	C34—C33—H33A	119.6
C14—As2—Ru2	116.77 (11)	C35—C34—C33	121.1 (5)
C20—As2—Ru2	117.12 (12)	C35—C34—H34A	119.4
C13—As2—Ru2	113.51 (11)	C33—C34—H34A	119.4
C38—P1—C26	102.54 (18)	C36—C35—C34	117.6 (4)
C38—P1—C32	103.69 (17)	C36—C35—C54	121.1 (4)
C26—P1—C32	101.05 (16)	C34—C35—C54	121.4 (5)
C38—P1—Ru3	114.60 (12)	C35—C36—C37	121.8 (4)
C26—P1—Ru3	116.66 (12)	C35—C36—H36A	119.1
C32—P1—Ru3	116.26 (13)	C37—C36—H36A	119.1
C6—C1—C2	118.4 (4)	C32—C37—C36	120.6 (4)
C6—C1—As1	124.8 (3)	C32—C37—H37A	119.7
C2—C1—As1	116.8 (3)	C36—C37—H37A	119.7
C3—C2—C1	120.7 (5)	C43—C38—C39	117.8 (4)
C3—C2—H2A	119.7	C43—C38—P1	122.3 (3)
C1—C2—H2A	119.7	C39—C38—P1	119.8 (3)
C4—C3—C2	120.1 (5)	C40—C39—C38	121.0 (4)
C4—C3—H3A	120.0	C40—C39—H39A	119.5
C2—C3—H3A	120.0	C38—C39—H39A	119.5
C3—C4—C5	120.4 (5)	C41—C40—C39	121.1 (4)
C3—C4—H4A	119.8	C41—C40—H40A	119.4
C5—C4—H4A	119.8	C39—C40—H40A	119.4
C4—C5—C6	119.2 (6)	C40—C41—C42	117.6 (4)
C4—C5—H5A	120.4	C40—C41—C55	122.0 (5)
C6—C5—H5A	120.4	C42—C41—C55	120.3 (5)
C1—C6—C5	121.3 (5)	C41—C42—C43	121.4 (4)
C1—C6—H6A	119.4	C41—C42—H42A	119.3
C5—C6—H6A	119.4	C43—C42—H42A	119.3
C12—C7—C8	118.3 (4)	C38—C43—C42	121.0 (4)
C12—C7—As1	122.6 (3)	C38—C43—H43A	119.5
C8—C7—As1	119.1 (3)	C42—C43—H43A	119.5
C7—C8—C9	121.6 (4)	O1—C44—Ru1	170.0 (4)
C7—C8—H8A	119.2	O2—C45—Ru1	175.8 (4)
C9—C8—H8A	119.2	O3—C46—Ru1	174.2 (4)
C10—C9—C8	119.3 (4)	O4—C47—Ru2	175.0 (4)
C10—C9—H9A	120.4	O5—C48—Ru2	176.5 (4)
C8—C9—H9A	120.4	O6—C49—Ru2	174.9 (4)
C9—C10—C11	119.9 (4)	O7—C50—Ru3	176.5 (4)
C9—C10—H10A	120.1	O8—C51—Ru3	177.7 (4)
C11—C10—H10A	120.1	O9—C52—Ru3	167.5 (4)
C10—C11—C12	120.9 (4)	C29—C53—H53A	109.5
C10—C11—H11A	119.6	C29—C53—H53B	109.5
C12—C11—H11A	119.6	H53A—C53—H53B	109.5
C7—C12—C11	120.0 (4)	C29—C53—H53C	109.5
C7—C12—H12A	120.0	H53A—C53—H53C	109.5
C11—C12—H12A	120.0	H53B—C53—H53C	109.5
As1—C13—As2	113.29 (16)	C35—C54—H54A	109.5
As1—C13—H13A	108.9	C35—C54—H54B	109.5
As2—C13—H13A	108.9	H54A—C54—H54B	109.5
As1—C13—H13B	108.9	C35—C54—H54C	109.5
As2—C13—H13B	108.9	H54A—C54—H54C	109.5
H13A—C13—H13B	107.7	H54B—C54—H54C	109.5
C19—C14—C15	118.0 (4)	C41—C55—H55A	109.5
C19—C14—As2	123.0 (3)	C41—C55—H55B	109.5
C15—C14—As2	119.0 (3)	H55A—C55—H55B	109.5
C16—C15—C14	120.6 (5)	C41—C55—H55C	109.5
C16—C15—H15A	119.7	H55A—C55—H55C	109.5
C14—C15—H15A	119.7	H55B—C55—H55C	109.5
			
C45—Ru1—Ru2—C48	−58.0 (6)	Ru1—As1—C1—C2	−62.2 (3)
C46—Ru1—Ru2—C48	−120.5 (4)	C6—C1—C2—C3	0.0 (7)
C44—Ru1—Ru2—C48	57.4 (4)	As1—C1—C2—C3	−179.6 (4)
As1—Ru1—Ru2—C48	149.8 (4)	C1—C2—C3—C4	0.2 (8)
Ru3—Ru1—Ru2—C48	−11.4 (4)	C2—C3—C4—C5	−0.7 (9)
C45—Ru1—Ru2—C47	36.8 (5)	C3—C4—C5—C6	1.0 (8)
C46—Ru1—Ru2—C47	−25.75 (17)	C2—C1—C6—C5	0.3 (7)
C44—Ru1—Ru2—C47	152.12 (17)	As1—C1—C6—C5	180.0 (4)
As1—Ru1—Ru2—C47	−115.48 (11)	C4—C5—C6—C1	−0.8 (8)
Ru3—Ru1—Ru2—C47	83.36 (11)	C1—As1—C7—C12	20.0 (4)
C45—Ru1—Ru2—C49	−139.9 (5)	C13—As1—C7—C12	−88.0 (3)
C46—Ru1—Ru2—C49	157.58 (16)	Ru1—As1—C7—C12	149.1 (3)
C44—Ru1—Ru2—C49	−24.55 (17)	C1—As1—C7—C8	−162.4 (3)
As1—Ru1—Ru2—C49	67.84 (11)	C13—As1—C7—C8	89.6 (3)
Ru3—Ru1—Ru2—C49	−93.32 (11)	Ru1—As1—C7—C8	−33.3 (4)
C45—Ru1—Ru2—As2	132.0 (5)	C12—C7—C8—C9	0.1 (6)
C46—Ru1—Ru2—As2	69.46 (12)	As1—C7—C8—C9	−177.5 (3)
C44—Ru1—Ru2—As2	−112.67 (12)	C7—C8—C9—C10	−0.6 (7)
As1—Ru1—Ru2—As2	−20.274 (16)	C8—C9—C10—C11	0.3 (8)
Ru3—Ru1—Ru2—As2	178.567 (15)	C9—C10—C11—C12	0.4 (8)
C45—Ru1—Ru2—Ru3	−46.6 (5)	C8—C7—C12—C11	0.6 (6)
C46—Ru1—Ru2—Ru3	−109.10 (12)	As1—C7—C12—C11	178.2 (3)
C44—Ru1—Ru2—Ru3	68.77 (12)	C10—C11—C12—C7	−0.9 (7)
As1—Ru1—Ru2—Ru3	161.159 (15)	C1—As1—C13—As2	93.4 (2)
C48—Ru2—Ru3—C51	−20.78 (19)	C7—As1—C13—As2	−162.9 (2)
C47—Ru2—Ru3—C51	67.13 (18)	Ru1—As1—C13—As2	−36.8 (2)
C49—Ru2—Ru3—C51	−114.50 (18)	C14—As2—C13—As1	−110.8 (2)
As2—Ru2—Ru3—C51	159.38 (14)	C20—As2—C13—As1	143.6 (2)
Ru1—Ru2—Ru3—C51	163.00 (13)	Ru2—As2—C13—As1	16.9 (2)
C48—Ru2—Ru3—C52	−120.56 (19)	C20—As2—C14—C19	74.5 (3)
C47—Ru2—Ru3—C52	−32.66 (18)	C13—As2—C14—C19	−30.8 (3)
C49—Ru2—Ru3—C52	145.72 (18)	Ru2—As2—C14—C19	−156.5 (3)
As2—Ru2—Ru3—C52	59.59 (13)	C20—As2—C14—C15	−105.4 (3)
Ru1—Ru2—Ru3—C52	63.21 (13)	C13—As2—C14—C15	149.3 (3)
C48—Ru2—Ru3—C50	73.68 (18)	Ru2—As2—C14—C15	23.6 (4)
C47—Ru2—Ru3—C50	161.58 (18)	C19—C14—C15—C16	1.0 (7)
C49—Ru2—Ru3—C50	−20.05 (18)	As2—C14—C15—C16	−179.1 (4)
As2—Ru2—Ru3—C50	−106.17 (13)	C14—C15—C16—C17	0.8 (8)
Ru1—Ru2—Ru3—C50	−102.55 (12)	C15—C16—C17—C18	−1.2 (9)
C48—Ru2—Ru3—Ru1	176.22 (13)	C16—C17—C18—C19	−0.3 (9)
C47—Ru2—Ru3—Ru1	−95.87 (13)	C17—C18—C19—C14	2.2 (8)
C49—Ru2—Ru3—Ru1	82.50 (12)	C15—C14—C19—C18	−2.5 (6)
As2—Ru2—Ru3—Ru1	−3.62 (4)	As2—C14—C19—C18	177.6 (3)
C45—Ru1—Ru3—C51	138.8 (3)	C14—As2—C20—C25	−172.9 (4)
C46—Ru1—Ru3—C51	44.2 (3)	C13—As2—C20—C25	−66.0 (4)
C44—Ru1—Ru3—C51	−134.4 (3)	Ru2—As2—C20—C25	58.3 (4)
As1—Ru1—Ru3—C51	−64.5 (2)	C14—As2—C20—C21	10.7 (4)
Ru2—Ru1—Ru3—C51	−29.6 (2)	C13—As2—C20—C21	117.7 (4)
C45—Ru1—Ru3—C52	63.9 (2)	Ru2—As2—C20—C21	−118.1 (3)
C46—Ru1—Ru3—C52	−30.7 (2)	C25—C20—C21—C22	−2.8 (7)
C44—Ru1—Ru3—C52	150.7 (2)	As2—C20—C21—C22	173.5 (4)
As1—Ru1—Ru3—C52	−139.41 (14)	C20—C21—C22—C23	0.7 (9)
Ru2—Ru1—Ru3—C52	−104.50 (14)	C21—C22—C23—C24	2.0 (11)
C45—Ru1—Ru3—C50	−109.03 (19)	C22—C23—C24—C25	−2.6 (11)
C46—Ru1—Ru3—C50	156.34 (19)	C21—C20—C25—C24	2.2 (8)
C44—Ru1—Ru3—C50	−22.24 (18)	As2—C20—C25—C24	−174.2 (5)
As1—Ru1—Ru3—C50	47.64 (13)	C23—C24—C25—C20	0.5 (10)
Ru2—Ru1—Ru3—C50	82.55 (13)	C38—P1—C26—C31	−73.2 (4)
C45—Ru1—Ru3—P1	−12.55 (15)	C32—P1—C26—C31	33.7 (4)
C46—Ru1—Ru3—P1	−107.18 (14)	Ru3—P1—C26—C31	160.7 (3)
C44—Ru1—Ru3—P1	74.23 (14)	C38—P1—C26—C27	102.0 (4)
As1—Ru1—Ru3—P1	144.11 (4)	C32—P1—C26—C27	−151.1 (3)
Ru2—Ru1—Ru3—P1	179.02 (3)	Ru3—P1—C26—C27	−24.1 (4)
C45—Ru1—Ru3—Ru2	168.42 (15)	C31—C26—C27—C28	1.5 (6)
C46—Ru1—Ru3—Ru2	73.79 (14)	P1—C26—C27—C28	−173.9 (4)
C44—Ru1—Ru3—Ru2	−104.80 (14)	C26—C27—C28—C29	−2.1 (7)
As1—Ru1—Ru3—Ru2	−34.91 (2)	C27—C28—C29—C30	1.0 (7)
C45—Ru1—As1—C1	97.1 (2)	C27—C28—C29—C53	179.1 (4)
C46—Ru1—As1—C1	−172.78 (19)	C28—C29—C30—C31	0.7 (7)
C44—Ru1—As1—C1	4.23 (19)	C53—C29—C30—C31	−177.4 (4)
Ru2—Ru1—As1—C1	−89.97 (14)	C29—C30—C31—C26	−1.2 (7)
Ru3—Ru1—As1—C1	−60.41 (14)	C27—C26—C31—C30	0.1 (6)
C45—Ru1—As1—C7	−23.72 (18)	P1—C26—C31—C30	175.5 (3)
C46—Ru1—As1—C7	66.35 (18)	C38—P1—C32—C33	−172.6 (3)
C44—Ru1—As1—C7	−116.65 (17)	C26—P1—C32—C33	81.4 (3)
Ru2—Ru1—As1—C7	149.15 (11)	Ru3—P1—C32—C33	−45.9 (4)
Ru3—Ru1—As1—C7	178.72 (11)	C38—P1—C32—C37	10.7 (4)
C45—Ru1—As1—C13	−138.62 (18)	C26—P1—C32—C37	−95.3 (4)
C46—Ru1—As1—C13	−48.55 (18)	Ru3—P1—C32—C37	137.4 (3)
C44—Ru1—As1—C13	128.46 (18)	C37—C32—C33—C34	−1.4 (6)
Ru2—Ru1—As1—C13	34.26 (12)	P1—C32—C33—C34	−178.3 (3)
Ru3—Ru1—As1—C13	63.83 (13)	C32—C33—C34—C35	0.7 (7)
C48—Ru2—As2—C14	−50.20 (18)	C33—C34—C35—C36	0.7 (7)
C47—Ru2—As2—C14	−139.99 (18)	C33—C34—C35—C54	−179.6 (4)
C49—Ru2—As2—C14	43.38 (18)	C34—C35—C36—C37	−1.3 (7)
Ru3—Ru2—As2—C14	129.64 (13)	C54—C35—C36—C37	179.0 (4)
Ru1—Ru2—As2—C14	126.47 (12)	C33—C32—C37—C36	0.8 (6)
C48—Ru2—As2—C20	71.2 (2)	P1—C32—C37—C36	177.5 (3)
C47—Ru2—As2—C20	−18.6 (2)	C35—C36—C37—C32	0.6 (7)
C49—Ru2—As2—C20	164.7 (2)	C26—P1—C38—C43	−19.2 (3)
Ru3—Ru2—As2—C20	−108.99 (16)	C32—P1—C38—C43	−124.1 (3)
Ru1—Ru2—As2—C20	−112.17 (16)	Ru3—P1—C38—C43	108.2 (3)
C48—Ru2—As2—C13	−170.87 (18)	C26—P1—C38—C39	165.4 (3)
C47—Ru2—As2—C13	99.34 (18)	C32—P1—C38—C39	60.5 (3)
C49—Ru2—As2—C13	−77.29 (17)	Ru3—P1—C38—C39	−67.3 (3)
Ru3—Ru2—As2—C13	8.97 (13)	C43—C38—C39—C40	1.3 (6)
Ru1—Ru2—As2—C13	5.80 (12)	P1—C38—C39—C40	176.9 (3)
C51—Ru3—P1—C38	−158.16 (19)	C38—C39—C40—C41	−1.3 (6)
C52—Ru3—P1—C38	−58.46 (18)	C39—C40—C41—C42	0.8 (6)
C50—Ru3—P1—C38	107.27 (18)	C39—C40—C41—C55	−177.9 (4)
Ru1—Ru3—P1—C38	5.28 (14)	C40—C41—C42—C43	−0.2 (6)
C51—Ru3—P1—C26	−38.36 (19)	C55—C41—C42—C43	178.5 (4)
C52—Ru3—P1—C26	61.34 (19)	C39—C38—C43—C42	−0.7 (5)
C50—Ru3—P1—C26	−132.93 (19)	P1—C38—C43—C42	−176.2 (3)
Ru1—Ru3—P1—C26	125.07 (14)	C41—C42—C43—C38	0.2 (6)
C51—Ru3—P1—C32	80.77 (19)	C45—Ru1—C44—O1	−52 (2)
C52—Ru3—P1—C32	−179.52 (19)	As1—Ru1—C44—O1	50 (2)
C50—Ru3—P1—C32	−13.79 (19)	Ru2—Ru1—C44—O1	141 (2)
Ru1—Ru3—P1—C32	−115.79 (14)	Ru3—Ru1—C44—O1	−162 (2)
C7—As1—C1—C6	−112.1 (4)	C51—Ru3—C52—O9	49.9 (18)
C13—As1—C1—C6	−9.9 (4)	C50—Ru3—C52—O9	−136.1 (16)
Ru1—As1—C1—C6	118.2 (3)	P1—Ru3—C52—O9	−46.3 (18)
C7—As1—C1—C2	67.5 (3)	Ru2—Ru3—C52—O9	137.7 (18)
C13—As1—C1—C2	169.7 (3)	Ru1—Ru3—C52—O9	−165.5 (19)

### Hydrogen-bond geometry (Å, °)

**Table:**

*D*—H···*A*	*D*—H	H···*A*	*D*···*A*	*D*—H···*A*
C27—H27A···O9	0.93	2.57	3.317 (5)	138
C54—H54A···O2^i^	0.96	2.60	3.306 (6)	131

Symmetry codes: (i) −*x*+1, *y*−1/2, −*z*+1/2.
